# Verbal autopsy and global mortality statistics: if not now, then when?

**DOI:** 10.1186/1478-7954-9-20

**Published:** 2011-07-27

**Authors:** Philip W Setel

**Affiliations:** 1 Measurement Learning and Evaluation for Global Health, Bill & Melinda Gates Foundation, PO Box 23350, Seattle, WA 98102, USA

## 

More than a decade ago, the World Health Organization pointed out the degree to which deficits in the production, sharing, and use of critical health information hampered evidence-based health development in countries with the poorest health status [1]. This "information paradox" in global health came to refer to the predicament in which countries with the greatest need for timely, accurate, and comprehensive health information - including on causes of death at the population level - have had the least access to it [2,3]. Since that time, some (but not enough) improvements in information systems and technologies have begun to fill voids in our knowledge of population health [4,5]. Throughout this period, however, comparatively little attention has been paid to advancing the science and practice of direct measurement of mortality and its causes - particularly among adults [6-11].

In 2000, the state of knowledge on verbal autopsy (VA, a term that covers the design and application of postmortem caregiver interviews, procedures for assigning one or more probable causes of death, and the aggregation and tabulation of population-level mortality statistics based on this data source) centered on a small group of demographers and epidemiologists, many of whom ran intervention trials in various demographic surveillance sites. Almost the entire community of scholarship was on a first-name basis; we could easily gather in a medium-sized conference room, and any of our students or colleagues could become an expert on the VA literature with a week or two of focused reading. Throughout this period, those who remained dedicated to maximizing the potential of VA made steady progress. Yet throughout, a deep and sometimes reflexive scepticism remained that VA could ever *really *deliver the goods as a reliable measurement tool. The persistent shortcomings in cause of death data, and reluctance to widely embrace VA outside of demographic surveillance sites, have forced the global health community to make do with sources of limited coverage and dubious quality and consistency, applying increasingly complex statistical analyses to "correct" for all manner of bias and nonsampling error.

The papers in this issue of *Population Health Metrics *go far in addressing central questions about how much VA can contribute to our measurement of health and health impact. How close to truth can VA ever get? How good is "good enough" for decision-making? Is our putative "gold standard" of medically certified deaths all that robust to begin with - in industrialized or lower-income countries? Can we make the production of VA data better, faster, and cheaper? What alternatives to demographic surveillance systems exist to permit the collection of mortality data from large, representative population samples? Can VA detect disease outbreaks, the population effects of antiretroviral therapy scale-up, and long-term trends in causes of death?

Collectively, this special issue should just about put to rest the conventional wisdom that in the 21st century those living on the margins of the global economy must continue to make do with models and guesstimates about leading causes of death for priority-setting and decision-making. This is not to deny that an important implementation research agenda remains. For example, there is an urgent need to identify optimal platforms, systems, and sample sizes for administration of VA, and to understand if and how VA-based death registration and cause of death statistics might be a stepping stone to increasing the coverage of functioning civil registration and death certification systems. While there have been a few examples of VA being administered on a large scale as an explicit part of the development of national statistics, such as the national causes of death rider survey to the 2007 Mozambique national census [12], they have been one-offs and not widely published.

Those who tout the impact of development assistance for health by confidently proclaiming the numbers of lives they have saved due to their investments - especially from particular diseases - rely on modeled estimates that are far less than the state of the art in observational epidemiology and medical demography. Part of the solution is for the international health sector to move on from the question of *whether *VA can be used to *how *we will use it to its maximum potential.

New advances in automated assignment of probable causes of death, such as those described in this series, combined with smartphone and tablet computing technology and the opportunity to further streamline VA interviews, promise to remove the remaining obstacles to meeting a far higher standard of credible evidence: to measure impact rather than just continue to model it. These game-changing innovations open the door ever wider to a future in which no one will go uncounted, and all lives will be more equally valued. Will this be the decade when we finally help those who die unseen to finally be seen and documented? Will we now start to support information systems capable of providing direct measurement of births, deaths, and causes of death among the most marginalized populations whose lives and deaths currently leave no trace in any official record or statistic?

## Competing interests

As indicated in the acknowledgments of the relevant articles, the Bill & Melinda Gates Foundation funded some of the research and findings presented in this issue of *Population Health Metrics*.
